# Dimanganese decacarbonyl catalyzed visible light induced ambient temperature depolymerization of poly(methyl methacrylate)

**DOI:** 10.1080/15685551.2022.2135730

**Published:** 2022-10-17

**Authors:** Zeynep Arslan, Hüseyin Cem Kiliclar, Yusuf Yagci

**Affiliations:** Department of Chemistry, Istanbul Technical University, Maslak, Turkey

**Keywords:** Visible light, dimanganese decacarbonyl, poly(methyl methacrylate), depolymerization

## Abstract

Recent years have witnessed an enormous development in photoinduced systems, opening up possibilities for advancements in industry and academia in terms of green chemistry providing environmentally friendly conditions and spatiotemporal control over the reaction medium. A vast number of research have been conducted on photoinduced systems focusing on the development of new polymerization methods, although scarcely investigated, depolymerization of the synthesized polymers by photochemical means is also possible. Herein, we provide a comprehensive study of visible light induced dimanganese decacarbonyl (Mn_2_(CO)_10_) assisted depolymerization system for poly(methyl methacrylate) with chlorine chain end prepared by Atom Transfer Radical Polymerization. Contrary to the conventional procedures demanding high temperatures, the approach offers ambient temperature for the photodepolymerization process. This novel light-controlled concept is easily adaptable to macroscales and expected to promote further research in the fields matching with the environmental concerns.

## 1. Introduction

Poly(methyl metacrylate) (PMMA) is a widely used commodity plastic that combines high optical clarity, impact strength and scratch resistance with lightweight nature [1–4]. Applications of PMMA including coatings [5,6], adhesives [7], ink [8] and lithography [9,10], directly benefit from mentioned physical characteristics. The consumption is expected to reach up to 4 M tons per year at 2027 [11] which bears the possibility of excessive waste production that highlights the necessity of recycling which has always been a problem, due to lack of eco-friendly approaches [12]. Today, approximately 6% of the produced polymer wastes are managed to be recycled [13]. Because of inefficient approaches, implementing sustainable recycling methods could be a struggle. PMMA cannot be easily depolymerized by hydrolysis or transesterification as they contain carbon–carbon bonds through the polymer chain. However, recent studies have examined the depolymerization of polymethacrylate derivatives demanding elevated temperatures about 300°C and toxic chemicals [14].

Recent advances on photochemical approaches provided environmentally friendly conditions for several polymerization reactions since the energy provided by light is used to induce polymerization reactions rather than thermal energy [15,16]. However, this potential was scarcely investigated in the field of depolymerization. Photochemical routes facilitate spatiotemporal control [17] [18], with lesser requirements of chemicals and energy [19] compared to conventional methods. Due to the advantages stated above, photoinduced processes have received tremendous attention and many synthetic applications have been reported [20].

Dimanganese decacarbonyl (Mn_2_(CO)_10_) absorbs light in the visible region and upon irradiation in the presence of certain additives, many reactive species can be formed [21]. Mn_2_(CO)_10_ based photoactive systems are extensively used for the synthesis of wide range of macromolecular structures such as telechelic polymers [22], block [23] and graft [24] copolymers and hyperbranched polymers [25,26]. Successfully applied processes include free radical polymerization [27], Atom Transfer Radical Polymerization (ATRP) [28,29], cationic polymerization [30,31], radical coupling reaction [32] and step-growth polymerization [33] [34]. Herein, we report a novel depolymerization method for PMMA with chlorine chain end. The main objective of this research is to decrease the depolymerization temperature to ambient conditions using the efficient nature of light for achieving a safer and more energy-efficient recycling process.

**Scheme 1.**
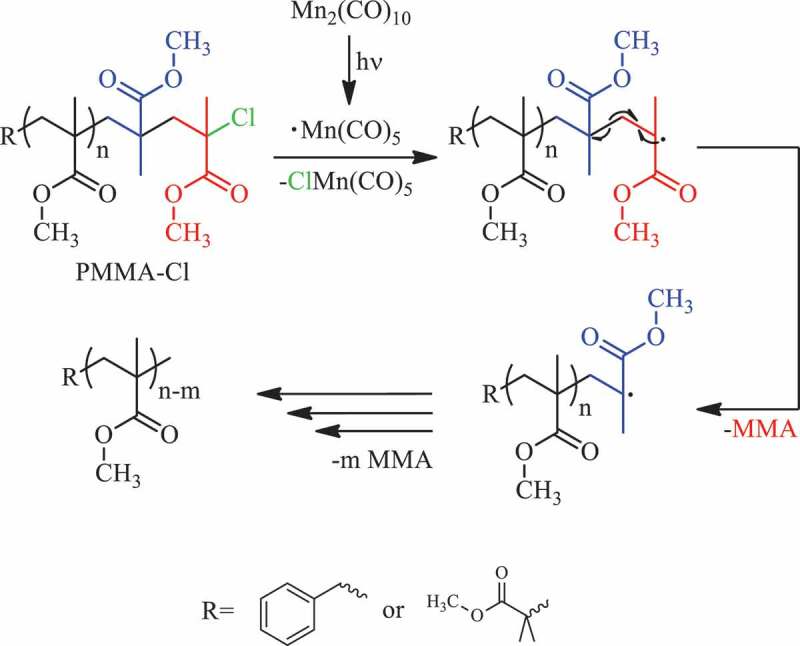
Visible light initiated unzipping type depropagation of PMMA-Cl with Mn_2_(CO)_10_ at ambient temperature.

Depolymerization is a process to shorten polymer chains and essentially yield monomers. The offered photodepolymerization mechanism is initiated by homolytic halogen abstraction at the chain end of the molecule and polymers with shorter chains are formed through depropagation enhanced by diluted reaction medium. (Scheme 1) The depropagation process is coined as the unzipping type as it starts from the chain end and continues through the polymer main chain [35].

## 2 Experimental

### 2.1 Materials

All reactions were performed under an inert atmosphere of nitrogen, using standard Schlenk techniques. Methyl methacrylate (Merck, 99%) was used after filtration through basic alumina. Toluene (Aldrich, 99.7%) and hexane (Aldrich, 98%) were dried and purified. PMDETA (Aldrich, 99%), CuCl (Aldrich, 97%), CuBr (Aldrich, 98%), benzyl chloride (Aldrich, 99%), were used as purchased. Dimanganese decacarbonyl (Merck, 98%) was used after purification by sublimation.

### 2.2. Characterization

^1^H NMR spectra were recorded on an Agilent VNMRS 500 NMR spectrometer system at room temperature in CDCl3 with Si(CH_3_)_4_ as an internal standard. On a TOSOH EcoSEC GPC system equipped with an autosampler system, a temperature-controlled pump, a column oven, a refractive index (RI) detector, a purge and degasser unit, and a TSK gel superhZ2000 4.6 mm ID × 15 cm × 2 cm column, gel permeation chromatography analysis were carried out. 1.0 mL.min^−1^ of tetrahydrofuran was utilized as the eluent at a temperature of 40°C. Calibrations were done by polystyrene standards with a limited molecular-weight distribution. By Eco-SEC analysis software, the data were analyzed.

### 2.3. Methods

PMMA derivatives having different molecular weights were synthesized by using the atomic transfer radical polymerization (ATRP) technique.

To obtain PMMA derivatives, toluene (5.35 mL), MMA (5.25 mL), PMDETA (72.5 μL), CuCl (50 mg) or CuBr (70 mg) to define the end group and benzyl chloride (38.5 μL) or benzyl bromide (55 μL) depending on the halogen of the copper salt were placed in a Schlenk tube in this order at ambient temperature under nitrogen atmosphere. Standard Schlenk techniques were applied to remove oxygen from reaction media. Polymerization was held at 90 ^o^C for 22 minutes to obtain PMMA-Cl^1^ 16 minutes to obtain both PMMA-Cl^2^ and PMMA-Br. (PMMA-Cl^1^)

Synthesis of PMMA-*co-*PGMA-Cl: toluene (10,7 mL), MMA (10 mL), GMA (0,66 mL) PMDETA (145 μL), CuCl (100 mg) and α-chloroethylbutyrate (125 μL) were placed in a Schlenk tube in this order at ambient temperature under nitrogen atmosphere. Standard Schlenk techniques were applied to remove oxygen from reaction media. Polymerization was held at 90℃ for 9 minutes. (PMMA-*co*-PGMA)

Depolymerization of PMMA derivatives: PMMA derivatives having different molecular weights were depolymerized under visible light. For this purpose, PMMA derivative (0,065 M), 12,4 mg Mn_2_(CO)_10_ and 6,7 mL toluene (*p*-dichlorobenzene for 170°C) were added to a Schlenk tube and standard Schlenk techniques were applied to remove oxygen from reaction media. Mixtures were irradiated in a photoreactor for up to 6 h in a variety of temperatures. GPC was chosen as the analysis tool as manganese impurities are interfering with ^1^H-NMR, MALDI ToF and gravimetric analyses.

## 3. Results and Discussion

Several PMMA derivatives with halogen chain end were synthesized by conventional ATRP for the subsequent depolymerization process. Initially, the effects of different halogens at the chain end were taken into consideration. Two types of PMMA, namely PMMA-Cl^1^ and PMMA-Br were synthesized. To be able to control the decrease of the molecular weight, coined as depolymerization yield, through ^1^H-NMR spectroscopy, benzyl chloride and benzyl bromide were chosen as ATRP initiators. The main differences to affect the depolymerization processes are the abstraction rate and nucleophilic characteristics of the halogen. Bromine at the chain end of PMMA is anticipated to be abstracted easily, yet increasing the radical concentration in the media to undergo undesired coupling reactions between polymer chains forming polymers without halide functionality [36]. According to the mentioned considerations, diluted reaction media without stirring was preferred. No depolymerization has been observed in dark conditions for any polymer samples.

**Figure 1.**
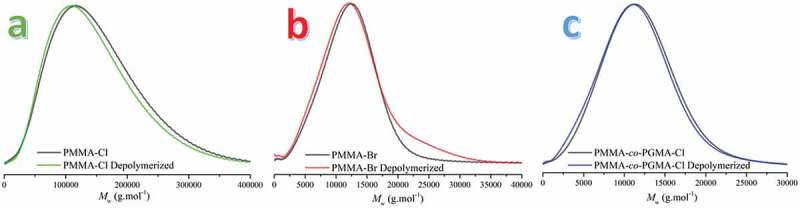
GPC chromatograms of a) PMMA-Cl and depolymerized PMMA-Cl b) PMMA-Br and depolymerized PMMA-Br c) PMMA-*co*-PGMA-Cl and depolymerized PMMA-*co*-PGMA-Cl. Depolymerizations were conducted by irradiation under 400 nm visible light at ambient temperature.

Even though depolymerization of PMMA-Br to some extent is noted in the chromatogram, longer polymer chains are formed as a consequence of coupling after bromine abstraction. Thus, the peaks are broadened and dispersity (*Đ*) is increased (Table 1 and Figure 1). On the other hand, PMMA-Cl^1^ undergoes a more regulated generation of radicals that catalyzes depolymerization rather than coupling reactions resulting 20% depolymerization yield.

**Table 1.** Molecular weight characteristics of PMMA-Cl^1^, PMMA-Br and PMMA-*co-*PGMA-Cl before and after visible light irradiation^a.^

Polymer	*M*_n_^b^ (g.mol^−1^)		*Đ*^b^	
	Before	After	Before	After
PMMA-Cl^1^	67,000	53,000	1,4	1,4
PMMA-Br	5400	5400	1,2	1,3
PMMA-*co-*PGMA-Cl	5100	4600	1,2	1,3

a- [Mn_2_(CO)_10_]:[Polymer]:[Tol.] = 1:2:2300 irradiated at 400 nm for 6 h. b-Determined by GPC using polystyrene standards.

To investigate the possibility of the application of the method to structurally different PMMA derivatives, chlorine end functional poly(methyl methacrylate-*co*-glycidyl methacrylate) (PMMA-*co*-PGMA-Cl) were synthesized. This polymer possesses epoxide rings in the side chain which remain unaffected during the radical depolymerization process. Comparable results were obtained at the same time interval.

**Figure 2.**
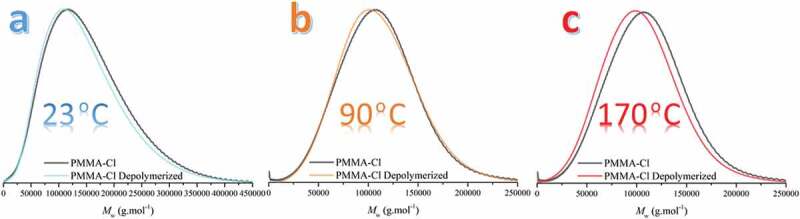
GPC chromatograms of PMMA-Cl and depolymerized PMMA-Cl under 400 nm visible light and a) 23°C b) 90°C c) 170°C.

Furthermore, it is a well-known fact that temperature has a major role in depolymerization [37,38]. Thus, the aim of the research is to decrease the requirement for elevated temperatures by implementing visible light. Therefore, the depolymerization process at 23℃, 90℃ and 170°C was investigated. To reach high temperatures, *p*-dichlorobenzene (*p*-DCB) was used as solvent (Table 2 and Figure 2).

**Table 2.** Molecular weight characteristics of PMMA-Cl before and after visible light irradiation^a.^

Polymer	Temp.(^o^C)	*M*_n_^c^(g.mol^−1^)		*Đ*^c^	
		Before	After	Before	After
PMMA-Cl^1 a^	23	67,000	53,000	1.4	1.4
PMMA-Cl^2 a^	90	56,000	41,000	1.3	1.7
PMMA-Cl^2 b^	170	56,000	33,000	1.3	2.0

a- [Mn_2_(CO)_10_]:[Polymer]:[Tol.] = 1:2:2300 irradiated at 400 nm for 6 h. b- [Mn_2_(CO)_10_]:[Polymer]:[*p*-DCB] = 1:2:2300 irradiated at 400 nm for 6 h. c-Determined by GPC using polystyrene standards.

**Figure 3.**
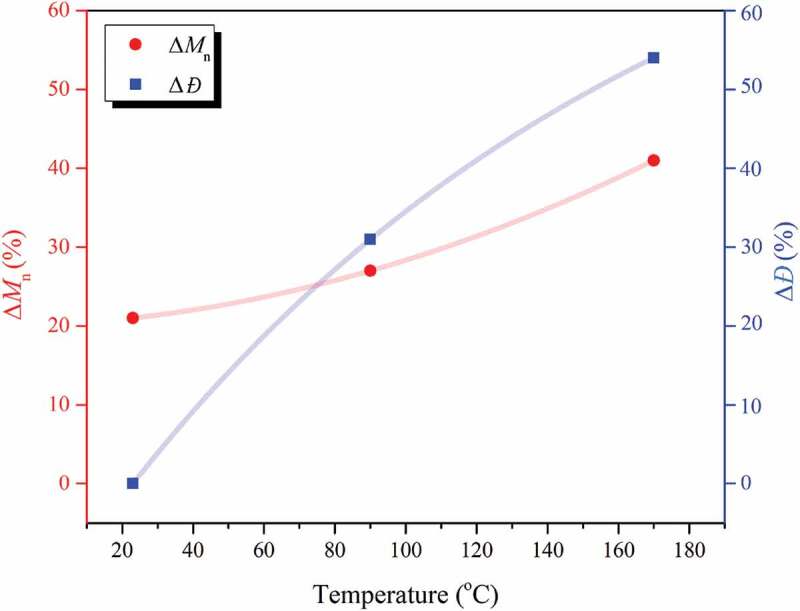
The effect of temperature on the *M*_n_ and *Đ* values obtained by GPC analysis.

Similar *M*_n_ values were obtained after depolymerization at 23°C and 90°C as indicated in Table 2. However, a drastic dispersity differences up to 34% was observed. Increase of the temperature to 170°C results in significant decrease in the *M*_n_ and increase in the dispersity indicating a more favorable depolymerization conditions by heat. The effect of heat on the depolymerization is demonstrated in Figure 3. It should be pointed out that even though catalyzing effect of the temperature is observed, ambient temperature results are still competitive.

**Figure 4.**
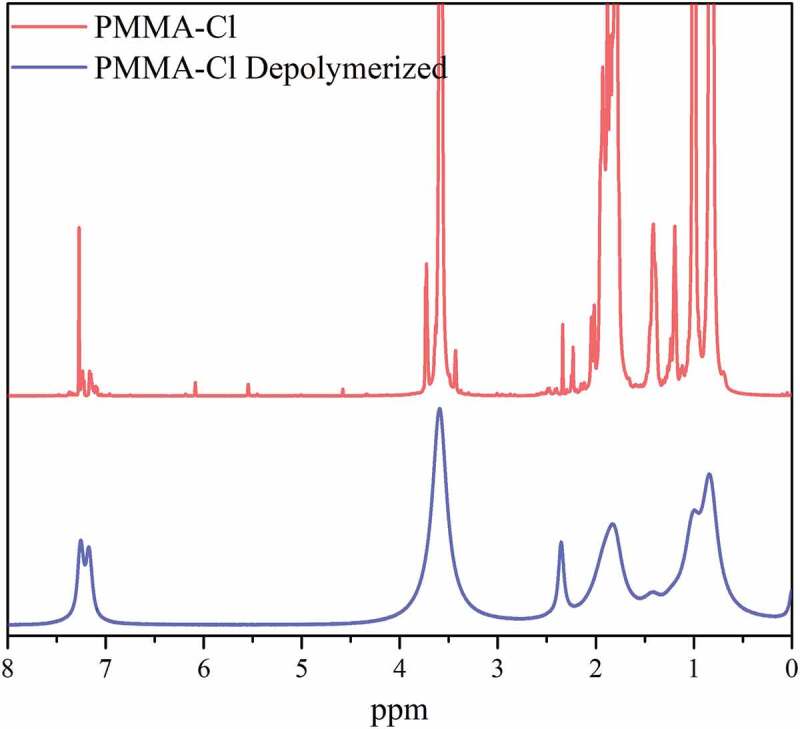
^1^H-NMR spectra of Bz-PMMA-Cl before and after depolymerized.

As mentioned above, benzyl chloride and benzyl bromide were chosen as ATRP initiators to control the depolymerization yield through ^1^H-NMR spectroscopy. The spectra of the initial polymer and the polymer after irradiation presented in Figure 4 further confirms the depolymerization, as the integration ratio of aromatic peaks of the benzylic chain end and peaks of the methoxy groups of PMMA increases through the process. Even though approximately 40% depolymerization can be estimated from ^1^H-NMR spectral analysis, the integration values are not extremely reliable due to the paramagnetic broadening effect of Mn_2_(CO)_10_. Detailed calculation is presented on Supporting Information.

## 4. Conclusion

This study reports on the detailed investigation on the visible light induced depolymerization of PMMA by using Mn_2_(CO)_10_ under ambient conditions. Depolymerization characteristics of PMMA polymers containing different halogens at the chain end were examined. While up to 20% depolymerization is achieved with PMMA-Cl, undesired chain extension by radical coupling reactions was observed in the depolymerization of PMMA-Br as a consequence of rapid production of radicals. Reaction kinetics of the process under identical irradiation conditions at 400 nm were examined by increasing the temperature. Obtained polymers characteristics were defined by ^1^H-NMR and GPC analyses. The reported light-induced ‘photodepolymerization’ approach provides significant advantages compare to the conventional techniques because of less energy requirement, low toxicity, spatiotemporal control over the reaction medium and eco-friendly conditions. This approach is easily adaptable to macroscales and can be conveyed to the structurally different polymers. Further studies in this line are in progress and will be reported elsewhere.

## Supplementary Material

Supplemental Material
