# Supplementary material for: Dimanganese decacarbonyl catalyzed visible light induced ambient temperature depolymerization of poly(methyl methacrylate)
Source: Des Monomers Polym. 2022 Oct 17;25(1):271–6. doi: 10.1080/15685551.2022.2135730 (PMC9586668; doi:10.1080/15685551.2022.2135730)
Supplement: Supplemental Material [file TDMP_A_2135730_SM6575.docx]

**Supporting Information**

**Dimanganesedecacarbonyl Catalyzed Visible Light Induced Ambient Temperature Depolymerization of Poly(methyl methacrylate)**

Zeynep Arslan^1^**,** Hüseyin Cem Kılıçlar^1^, Yusuf Yağcı^1*^
^1^Istanbul Technical University, Faculty of Science, Department of Chemistry

Maslak, Istanbul, 34469, Turkey

[arslanze21@itu.edu.tr](mailto:arslanze21@itu.edu.tr) ; [kiliclar@itu.edu.tr](mailto:kiliclar@itu.edu.tr) ; [yusuf@itu.edu.tr](mailto:yusuf@itu.edu.tr)

Details of GPC studies.

To replicate the reaction conditions, prepolymer was reprecipitated from the reaction solvent and three GPC samples were prepared. All analyses were done using the same batch of THF. Measurements were performed on a TOSOH EcoSEC GPC system equipped with an auto-sampler system, a temperature-controlled pump, a column oven, a refractive index (RI) detector, a purge and degasser unit and a TSKgel superhZ2000, 4.6 mm ID × 15 cm × 2 cm column. Eluent used at a flow rate of 1.0 mL/min at 40 °C. The refractive index detector was calibrated with polystyrene standards having narrow molecular-weight distributions. The data were analyzed using Eco-SEC analysis software. Results are given in **Table S1**.

| **Table S1.** GPC report sheets of consecutive analyses of PMMA-Cl and PMMA-*co*-PGMA-Cl to measure the error. | | |
| --- | --- | --- |
|  | PMMA-Cl | PMMA-*co*-PGMA-Cl |
| 1 | 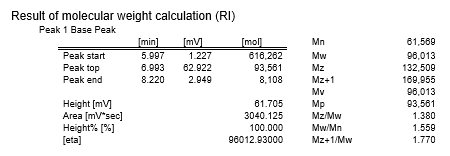 | 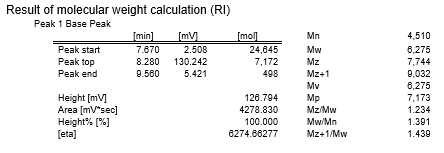 |
| 2 | 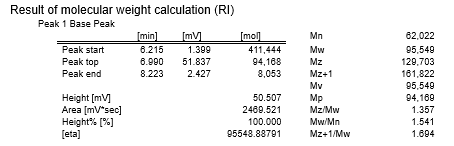 | 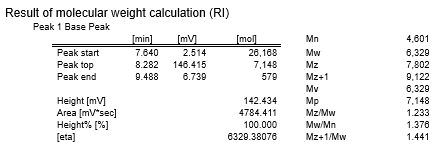 |
| 3 | 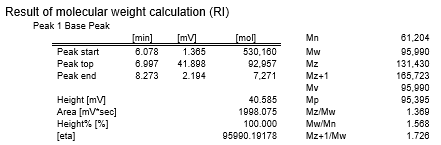 | 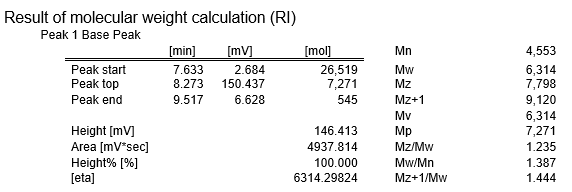 |

Standard deviations were found to be 334.6 g mol^-1^ for PMMA-Cl and 37.1 g mol^-1^ for PMMA-*co*-PGMA-Cl which are less than 1%.

Determining the reaction time for depolymerization

Reaction time was determined by aliquot sample gathering and measuring *M*_n_ decrease in predetermined time intervals. 6 h was determined to be appropriate and therefore applied to all experiments.

| **Table S2.** Visible light induced depolymerization of different polymers. | | | | | |
| --- | --- | --- | --- | --- | --- |
| Sample | Illumination Time (h) | *M*_n_ (g.mol^-1^) Before | *M*_n_  (g.mol^-1^) After | *Đ* Before | *Đ*  After |
| PMMA-*co-*PGMA-Cl | 3 | 5400 | 5200 | 1.2 | 1.2 |
| PMMA-*co-*PGMA-Cl | 6 | 5400 | 4700 | 1.2 | 1.3 |
| PMMA-*co-*PGMA-Cl | 12 | 5050 | 4600 | 1.2 | 1.3 |
| PMMA-Br | 12 | 5400 | 5400 | 1.2 | 1.3 |
| PMMA-Br | 18 | 5400 | 5400 | 1.2 | 1.3 |
| PMMA-Cl | 6 | 70000 | 62000 | 1.4 | 1.4 |
| PMMA-Cl | 8 | 70000 | 62000 | 1.4 | 1.4 |
|  |  |  |  |  |  |

Investigation of the effect of depolymerization on epoxide rings.

Epoxide rings appear 3 ppm before and after depolymerization in the ^1^H-NMR spectrum. Manganese residual impurity broadened the spectrum of depolymerized PMMA-*co*-PGMA-Cl due to the paramagnetic effect. (Figure S1)





Figure S1. ^1^H-NMR spectra of PMMA-*co*-PGMA-Cl (black) and depolymerized PMMA-*co*-PGMA (red).

Calculation of depolymerization using ^1^H-NMR.

As indicated by integration ratio of the intensities of benzylic hydrogens present at the chain end of the initial polymer and depolymerized sample, esteric methoxide hydrogen abundance is decreased 40% which can be attributed to depolymerization. (Figure S2, S3 and S4) Molecular weights were calculated using ^1^H-NMR spectra and tabulated in Table S1.


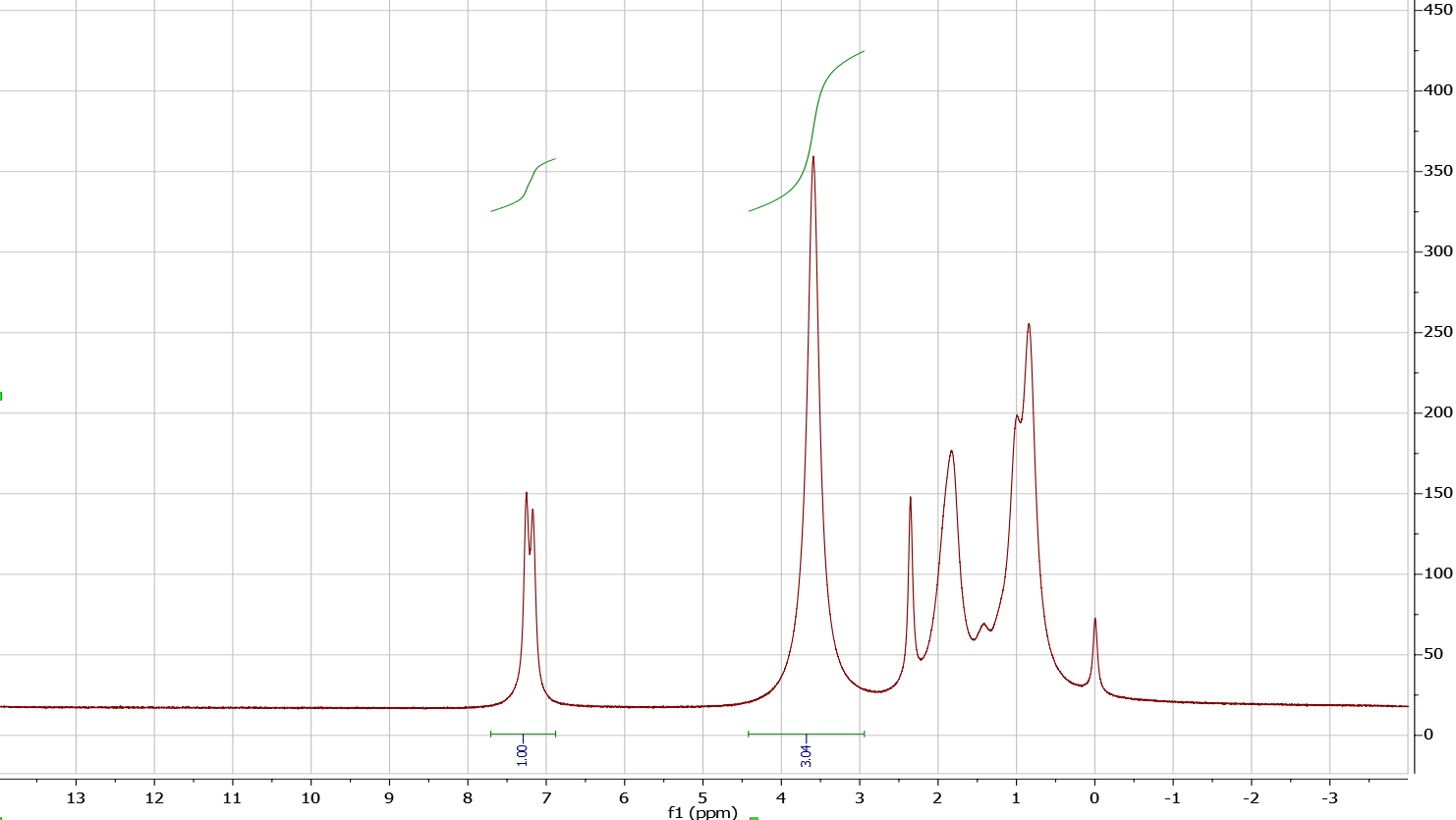


Figure S2. ^1^H-NMR spectrum of depolymerized Bz-PMMA- Cl


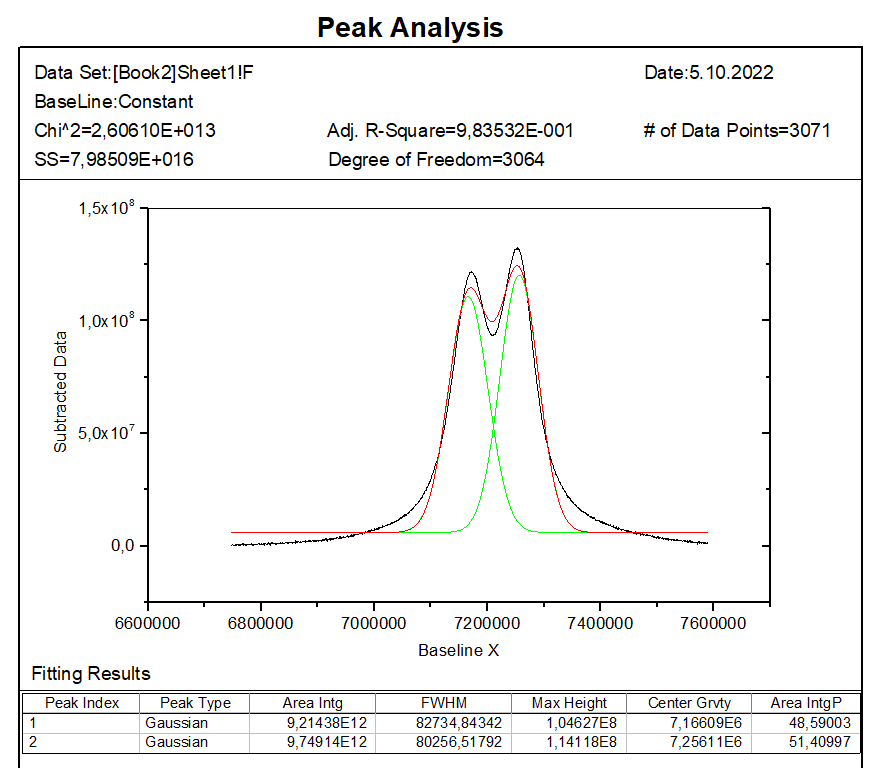


Figure S3. Peak analysis of ^1^H-NMR spectra of depolymerized Bz-PMMA- Cl to separate the integration values of CHCl_3_ and aromatic hydrogen peaks.


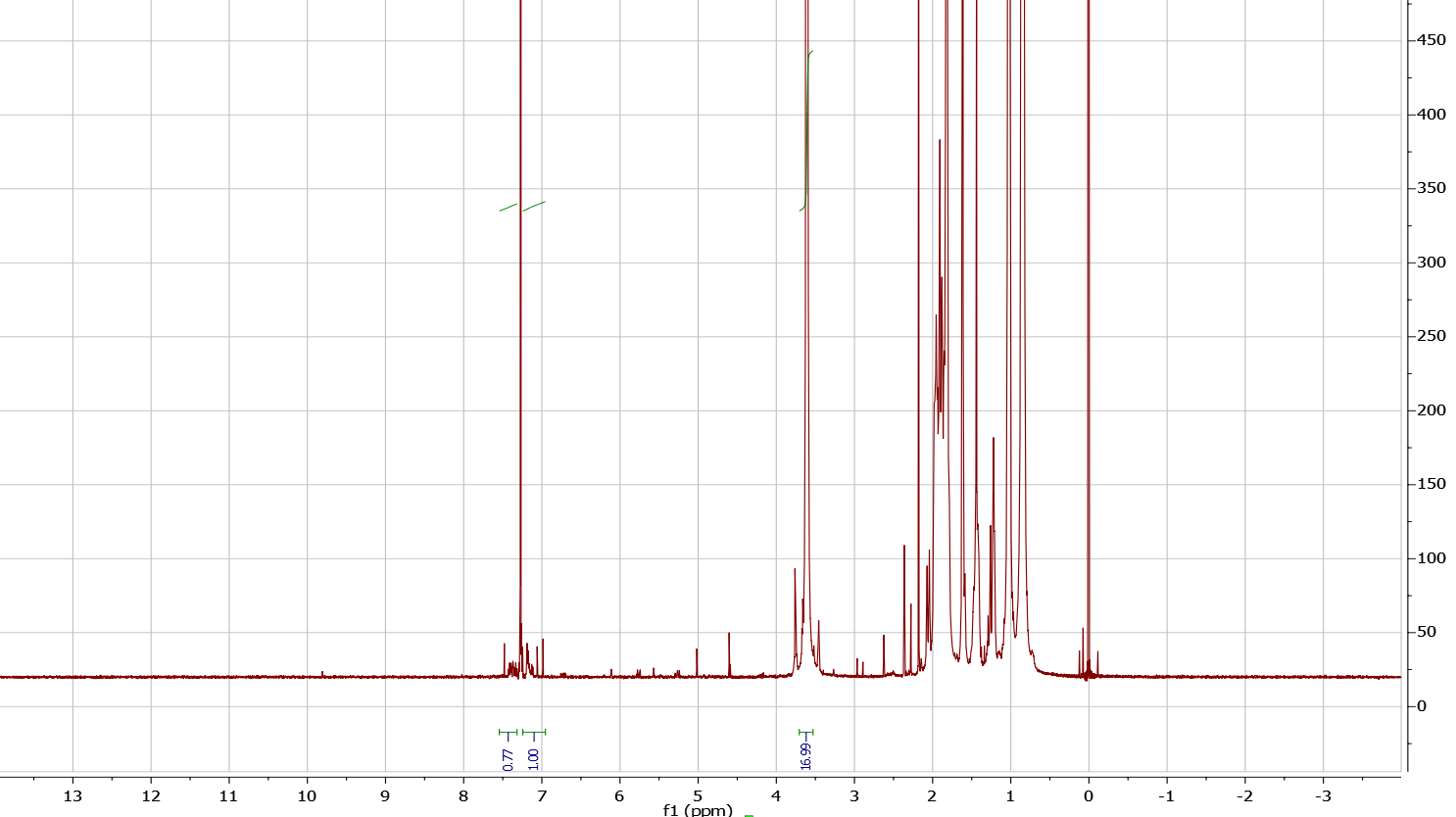


Figure S4. ^1^H-NMR spectrum of initial Bz-PMMA- Cl

| Table S1. Molecular weight decrease caused by depolymerization. | | |
| --- | --- | --- |
| Polymer | Molecular Weight Before ^a^  (g mol^-1^) | Molecular Weight After ^a^  (g mol^-1^) |
| Bz-PMMA-Cl | 1800 | 1100 |
| a- Calculated by ^1^H-NMR spectrum | | |

By calculation with ^1^H-NMR, it is observed that the initial polymer consists of up to 17 repeating units, 1 benzyl group and 1 chlorine at the chain end yielding approximately a molecular weight of 1800 g mol^-1^. After depolymerization, the composition of the polymer contains 10 repeating units and 1 benzyl group observable at the chain end yielding approximately a molecular weight of 1100 g mol^-1^.
